# Supplementary material for: Is shape in the eye of the beholder? Assessing landmarking error in geometric morphometric analyses on live fish
Source: PeerJ. 2023 Aug 17;11:e15545. doi: 10.7717/peerj.15545 (PMC10440062; doi:10.7717/peerj.15545)
Supplement: Supplemental Information 1 [file peerj-11-15545-s001.pdf]

Supplementary material

Supplementary Table 1. Operator classification obtained by Between-group PCA using the leave-one-out cross-validation. Numbers represent fish specimens assigned to operators based on their morphology. Percentage of classification accuracy is shown.

	**Op.1**	**Op.2**	**Op.3**	**Op.4**	**Overall classification accuracy**
**Op.1**	222	16	30	21	76.8 %
**Op.2**	15	267	3	4	92.4 %
**Op.3**	35	8	220	26	76.1 %
**Op.4**	19	4	16	250	86.5 %

Supplementary Table 2. River classification obtained by Between-group PCA for each operator using the leave-one-out cross-validation. Numbers represent specimens assigned to rivers based on their morphology. Percentage of classification accuracy is shown.

**Operator**	**River of origin**	**Assigned to Oykel**	**Assigned to Spey**	**Overall classification accuracy**
Op.1	Oykel	113	32	73.4%
	Spey	45	99	
Op.2	Oykel	106	39	70.9%
	Spey	45	99	
Op.3	Oykel	103	42	70.6%
	Spey	43	101	
Op.4	Oykel	97	48	68.5%
	Spey	43	101	

Supplementary Table 3. Pairwise tests of angles between body shape differences among rivers (Spey vs. Oykel) and operators (e.g., mean shape Op.1 - mean shape Op.2). Significant *P*-values (in bold) indicate that shape change vectors are similar to each other.

Comparison		*P*-value	Angle (degrees)
Op.1 (Spey vs. Oykel)	Op.1 - Op.2 (mean shapes)	0.227	83.1
Op.1 (Spey vs. Oykel)	Op.1 - Op.3 (mean shapes)	**0.026**	72.2
Op.1 (Spey vs. Oykel)	Op.1 - Op.4 (mean shapes)	**0.021**	71.4
Op.1 (Spey vs. Oykel)	Op.2 - Op.3 (mean shapes)	**0.034**	73.3
Op.1 (Spey vs. Oykel)	Op.2 - Op.4 (mean shapes)	**0.034**	73.3
Op.1 (Spey vs. Oykel)	Op.3 - Op.4 (mean shapes)	0.193	82.0
Op.2 (Spey vs. Oykel)	Op.1 - Op.2 (mean shapes)	0.425	88.3
Op.2 (Spey vs. Oykel)	Op.1 - Op.3 (mean shapes)	**0.048**	74.7
Op.2 (Spey vs. Oykel)	Op.1 - Op.4 (mean shapes)	**0.035**	73.3
Op.2 (Spey vs. Oykel)	Op.2 - Op.3 (mean shapes)	0.188	81.8
Op.2 (Spey vs. Oykel)	Op.2 - Op.4 (mean shapes)	0.128	79.5
Op.2 (Spey vs. Oykel)	Op.3 - Op.4 (mean shapes)	0.205	82.4
Op.3 (Spey vs. Oykel)	Op.1 - Op.2 (mean shapes)	0.345	86.3
Op.3 (Spey vs. Oykel)	Op.1 - Op.3 (mean shapes)	0.083	77.2
Op.3 (Spey vs. Oykel)	Op.1 - Op.4 (mean shapes)	0.110	78.7
Op.3 (Spey vs. Oykel)	Op.2 - Op.3 (mean shapes)	0.291	84.9
Op.3 (Spey vs. Oykel)	Op.2 - Op.4 (mean shapes)	0.267	84.3
Op.3 (Spey vs. Oykel)	Op.3 - Op.4 (mean shapes)	0.351	86.5
Op.4 (Spey vs. Oykel)	Op.1 - Op.2 (mean shapes)	0.345	86.3
Op.4 (Spey vs. Oykel)	Op.1 - Op.3 (mean shapes)	**0.049**	74.8
Op.4 (Spey vs. Oykel)	Op.1 - Op.4 (mean shapes)	**0.017**	70.5

Op.4 (Spey vs. Oykel)	Op.2 - Op.3 (mean shapes)	0.088	77.5
Op.4 (Spey vs. Oykel)	Op.2 - Op.4 (mean shapes)	**0.046**	74.6
Op.4 (Spey vs. Oykel)	Op.3 - Op.4 (mean shapes)	0.124	79.3

Supplementary Table 4. River classification obtained by Between-group PCA for merged datasets of operators 2-4 using the leave-one-out cross-validation. Numbers represent specimens assigned to rivers based on their morphology. Percentage of classification accuracy is shown.

**Operator**	**River of origin**	**Assigned to Oykel**	**Assigned to Spey**	**Overall classification accuracy**
Op.2	Oykel	133	12	92.7%
Op.4	Spey	9	135	
Op.4	Oykel	132	13	94.5%
Op.2	Spey	3	141	

Supplementary Table 5. Pairwise tests of angles between body shape differences among rivers detected by merged datasets of Ops. 2-4 as well as by the separate datasets of all four operators. Measurements of angles (degrees) between bwgPC1 vectors (below the diagonal) and *P*-values (above the diagonal) are shown. Significant *P*-values (in bold) indicate that shape change vectors are similar to each other.

	Op.2-Oykel Op.4-Spey	Op.2-Spey Op.4-Oykel	Op.1 (Oykel vs. Spey)	Op.2 (Oykel vs. Spey)	Op.3 (Oykel vs. Spey)	Op.4 (Oykel vs. Spey)
Op.2-Oykel Op.4-Spey		**1.4×10^{-6}**	0.445	0.093	**0.022**	0.304
Op.2-Spey Op.4-Oykel	48.7°		**3.0×10^{-7}**	**2.9×10^{-5}**	**0.001**	**4.4×10^{-7}**
Op.1 (Oykel vs. Spey)	88.7°	46.4°		**5.3×10^{-18}**	**1.8×10^{-14}**	**6.0×10^{-17}**
Op.2 (Oykel vs. Spey)	77.8°	54.2°	22.7°		**1.1×10^{-22}**	**1.9×10^{-19}**
Op.3 (Oykel vs. Spey)	71.5°	61.0°	28.4°	17.0°		**1.9×10^{-19}**
Op.4 (Oykel vs. Spey)	85.3°	46.9°	24.3 °	20.8°	20.8°	

Supplementary Table 6. Nested Procrustes ANOVA summary statistics of effect of fish specimen ('ID') and operators on shape variation after three repeated digitisations of twenty salmon smolts (Df = Degrees of freedom, SS = Sum of squares, *F* = *F* statistics, *Z* = Effect size).

	**Df**	**SS**	r^2	***F***	***Z***	***P*-value**
ID	19	0.062678	0.67295	80.8462	30.227	0.0001
ID:Operator	60	0.023932	0.25695	9.7753	14.920	0.0001
Residuals	160	0.006529	0.07010			
Total	239	0.093139				

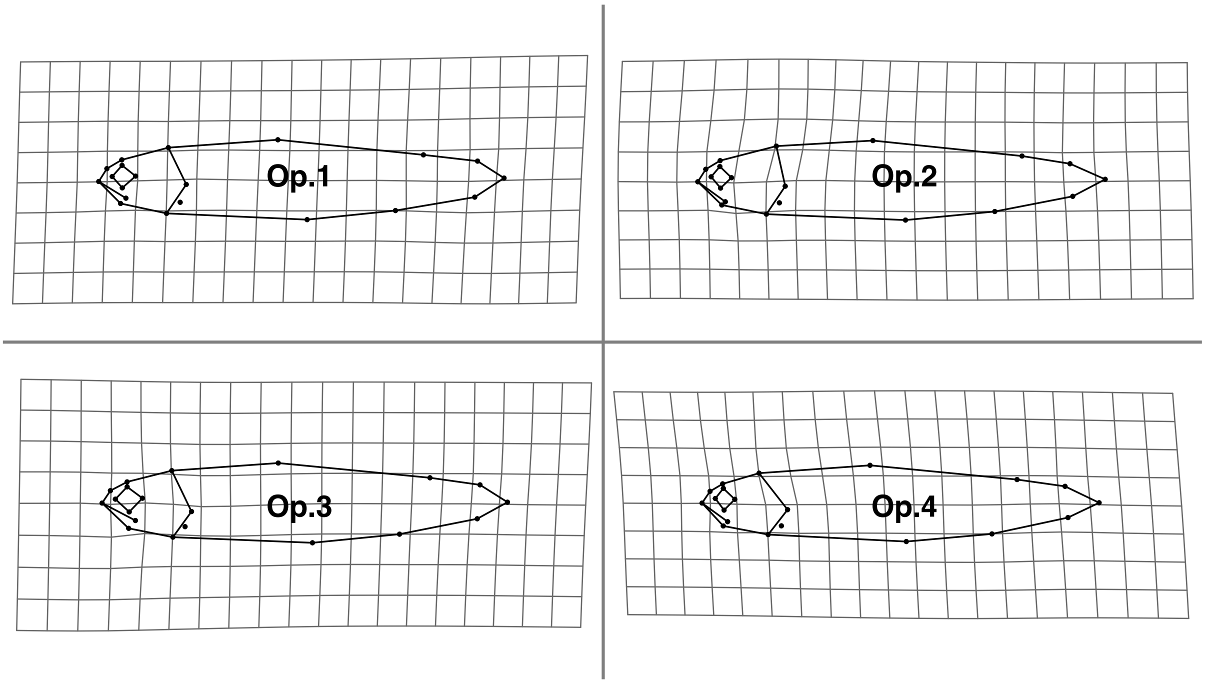

Supplementary Figure 1. Differences in shape between operators illustrated using thin-plate spline deformation grids. Mean body shapes for each operator are projected against the overall shape calculated for all specimens from all four operators. Morphological differences were magnified two times for easier visualisation.

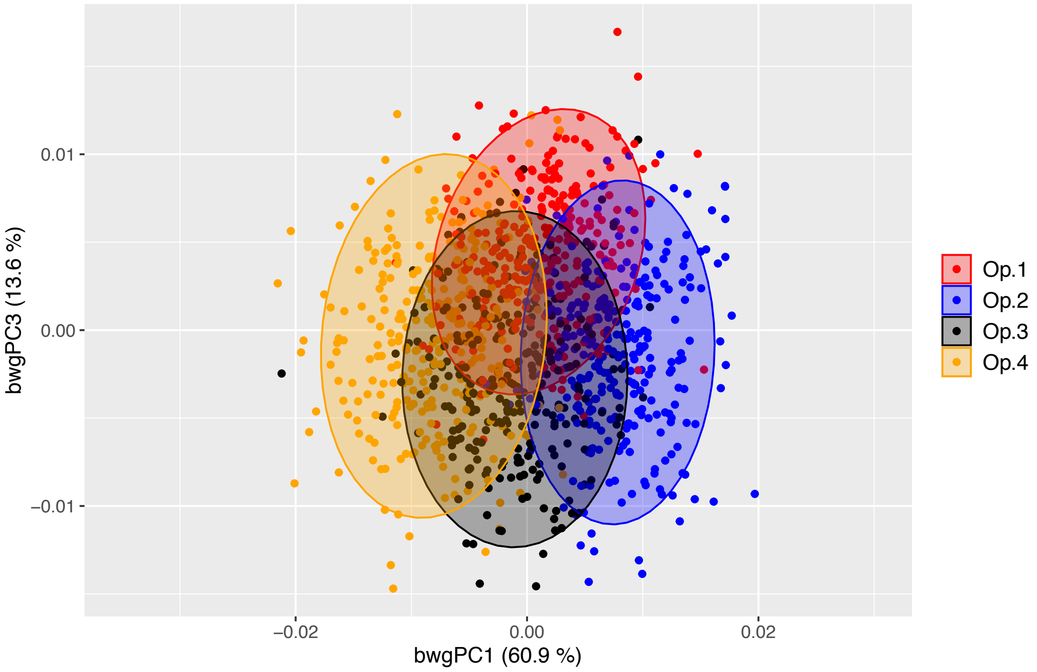

Supplementary Figure 2. Between-operator PCA scatterplot showing the cross-validated scores along the first and third between-group principal components (bwgPCs). Dots represent individual Atlantic salmon (n = 289) landmarked by four independent operators (different colours). Between-operator variance (%) explained by the first and third axes is shown.

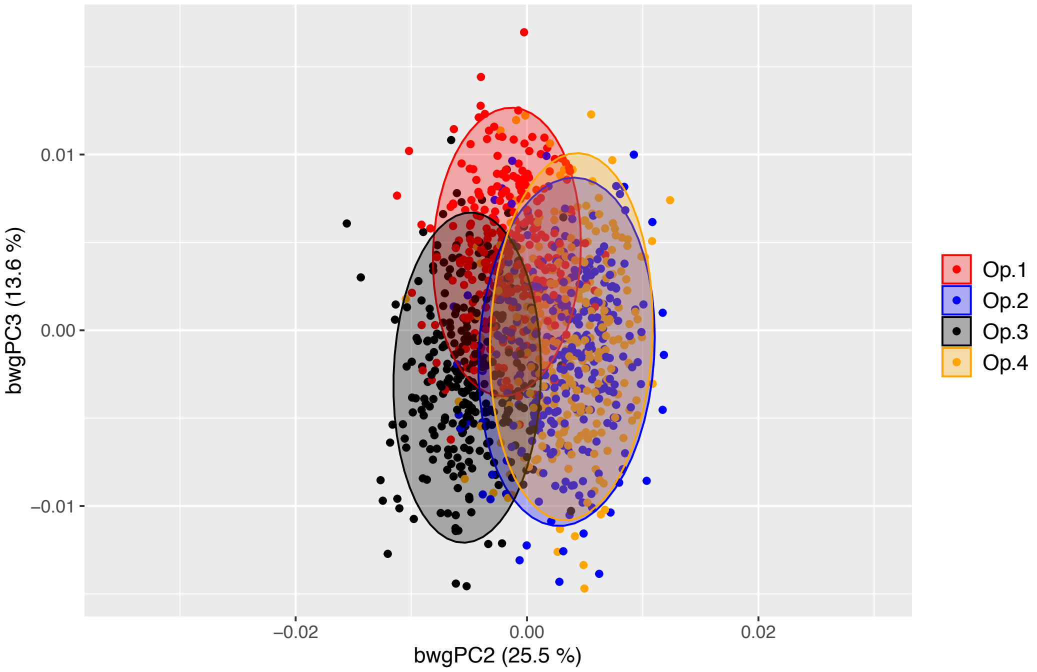

Supplementary Figure 3. Between-operator PCA scatterplot showing the cross-validated scores along the second and third between-group principal components (bwgPCs). Dots represent individual Atlantic salmon (n = 289) landmarked by four independent operators (different colours). Between-operator variance (%) explained by the second and third axes is shown.
